# On Maximizing the Lifetime of Wireless Sensor Networks by Optimally Assigning Energy Supplies

**DOI:** 10.3390/s130810219

**Published:** 2013-08-09

**Authors:** Rafael Asorey-Cacheda, Antonio Javier García-Sánchez, Felipe García-Sánchez, Joan García-Haro, Francisco Javier Gonzalez-Castaño

**Affiliations:** 1 Centro Universitario de la Defensa, University of Vigo, Praza de España, Marín 36920, Spain; 2 Departament of Information and Communication Technologies, Universidad Politécnica de Cartagena (UPCT), ETSIT, Campus Muralla del Mar 1, Cartagena 30202, Spain; E-Mails: antoniojavier.garcia@upct.es (A.J.G.-S.); felipe.garcia@upct.es (F.G.-S.); joang.haro@upct.es (J.G.-H.); 3 Departamento de Enxeñaría Telemática, University of Vigo, Campus, Vigo 36310, Spain; E-Mail: javier@det.uvigo.es

**Keywords:** wireless sensor networks, lifetime optimization, energy supply assignment

## Abstract

The extension of the network lifetime of Wireless Sensor Networks (WSN) is an important issue that has not been appropriately solved yet. This paper addresses this concern and proposes some techniques to plan an arbitrary WSN. To this end, we suggest a hierarchical network architecture, similar to realistic scenarios, where nodes with renewable energy sources (denoted as primary nodes) carry out most message delivery tasks, and nodes equipped with conventional chemical batteries (denoted as secondary nodes) are those with less communication demands. The key design issue of this network architecture is the development of a new optimization framework to calculate the optimal assignment of renewable energy supplies (primary node assignment) to maximize network lifetime, obtaining the minimum number of energy supplies and their node assignment. We also conduct a second optimization step to additionally minimize the number of packet hops between the source and the sink. In this work, we present an algorithm that approaches the results of the optimization framework, but with much faster execution speed, which is a good alternative for large-scale WSN networks. Finally, the network model, the optimization process and the designed algorithm are further evaluated and validated by means of computer simulation under realistic conditions. The results obtained are discussed comparatively.

## 1. Introduction

Large-scale wireless sensor networks (WSN) present important design challenges. Among them, network lifetime is a major topic of research, because this kind of network is usually intended for outdoor operation during long periods of time. Moreover, suitable planning before deployment is a fundamental task, albeit extremely complex, due to the many configurations that can be established. Thus, to extend WSN lifetime, we propose to provide certain nodes with additional power supplies (e.g., renewable energy sources). This paper addresses this idea and introduces some novel techniques, an optimization framework and a node assignment algorithm, to allow network planning before the deployment of arbitrary WSNs.

A Wireless Sensor Network consists of a large group of autonomous low-cost devices (nodes)distributed over an area that monitors one or several environmental variables of interest. In thesenetworks, the sources are nodes that capture and collaboratively dispatch the acquired data (temperature, humidity, lightness, *etc.*) to the sink node/s, which is/are usually out of their direct coverage. As a consequence, intermediate nodes, in the path between sources and sinks, are in charge of retransmitting the sensed data, selecting, in a distributed manner, the best path to the sink. WSNs can be used to observe the physical world at a very fine spatial and temporal granularity and, thus, enabling a plethora of innovative applications in environment monitoring, agriculture, industrial plants, domotic systems, *etc.*

Current node technology and network lifetime have restricted the deployment of WSNs in recent years. As many other autonomous electronic devices, the most common power source for WSN nodes (including the sensing instrumentation and radio communication subsystems) are chemical batteries. However, these batteries have two main drawbacks: (i) fast depletion when nodes operate intensively; and (ii) non-environmental sustainability of their chemical elements, since they can be abandoned in the monitoring area. To overcome these drawbacks, many real deployments replace chemical batteries with renewable energy supplies, e.g., solar panels or small windmills [1]. Depending on node location, these energy harvesting systems provide a long lifetime and, if necessary, continuous operation. However, renewable power supplies are mostly used in outdoor scenarios and notably increase the cost of the network deployment, compared with the regular battery case, especially when all nodes require them. Note that the node placement issue is out of the scope of this paper, as it is assumed that in most WSN deployments, nodes are placed where sensing tasks are required.

The utilization of renewable energy is limited by the network environmental conditions and its cost. To this aim, in this paper, we deal with a more realistic WSN deployment, which combines renewable and chemical power supplies in the same network as in [2,3]. Note that WSN nodes employ only one of them to reduce costs. Moreover, the decision to power the nodes with one type of energy or another is made in accordance with their operational needs. For instance, edge nodes, located far from the sink, should have shorter operation times, because they will not be used for neighbor message relaying. In this case, cheap and simple chemical batteries are still a suitable option. On the contrary, nodes closer to the sink node have to deliver messages from other nodes with high probability. Consequently, a renewable energy source may be a better option. This arrangement suggests a hierarchical network architecture, where nodes with renewable sources (denoted from now on as primary nodes) carry out most message delivery and nodes equipped with conventional batteries (denoted as secondary nodes) are those with less communication demands. The key design issue of this network architecture is the optimal selection of the proper number and node assignment with renewable energy means and, on the other hand, an estimate of the suitable number of hops between the source and sink. We face this as a planning problem, which is modeled and optimized within this paper.

The main contributions of this work are: First, we calculate the optimal assignment of primary nodes to maximize network lifetime, obtaining their number and node assignment. To solve this, we employ a linear programming model (LP) similar to [4]. Second, we evaluate the performance of the network, simultaneously providing the maximum lifetime and the minimum average number of hops to the sink. Again, it is modeled as an LP problem that, in turn, optimizes the flow assignment (traffic load balancing) among a node and its neighbors in coverage. Third, we present a novel algorithm that approaches the results of the optimization framework, but in a short execution time. Fourth, the network model, the optimization process and the algorithm design are further evaluated on an IEEE (Institute of Electrical and Electronics Engineers) 802.15.4-based scenario [5] by means of computer simulation. This last step validates the optimization and algorithm results and offers insight into a more realistic network given the planning objectives and for different network metrics.

Lifetime optimization provides information on how long all nodes in a network can operate, but it does not specify a set of routing features as problem constraints. In fact, for a calculated lifetime, there may exist multiple routing alternatives. From them, our proposal selects the path whose goal is the minimization of the number of hops between source and sink. In this sense, although *a priori* different suitable WSN routing schemes converge to this goal faster than the proposal presented here, the selection of a specific routing algorithm restricts the research under consideration to a particular case. In contrast, we provide a general case study, which employs two different types of energy supplies and, consequently, implies two roles for the network nodes (primary or secondary nodes) in order to maximize the network lifetime metric.

The rest of the paper is organized as follows. Section 2 reviews some relevant work on lifetime maximization approaches found in the recent WSN literature. Section 3 introduces the linear model and problem formulation, and Section 4 presents the novel primary node assignment algorithm. Section 5 discusses the numerical results obtained by both the linear programming model and the primary assignment algorithm. In Section 6, these optimization results are validated by the ones achieved through simulation. Finally, in Section 7, we conclude the paper.

## 2. Related Work

WSN lifetime maximization on energy harvesting systems has been tackled in the scientific literature from many different points of view. However, it can be broadly categorized into two groups of work. In particular, work included in the first group addresses lifetime maximization as an optimization of the energy consumption for a given network. The most commonly used optimization technique is LP. The second category of work also employs LP, but the main objectives are to appropriately determine the node's location in the network and/or the assignment of a particular role to some of those network nodes in order to increase the network lifetime. Under these general premises, in the next paragraphs, we highlight and describe the following relevant related work.

Concerning the first group of approaches, the work in [6] studies and optimizes the operation lifetime of a WSN consisting of nodes fed by solar-based renewable energy systems. Nodes may modify their tasks dynamically, according to external conditions. This implies a variation of the energy consumed and, therefore, the new estimation of the network lifetime. In addition, some of these tasks may involve a noticeable increase in node power consumption, which might make the energy supplied by the solar energy feeding system insufficient. In this case, a linear formulation technique is used to minimize the residual energy of nodes and when the time horizon (maximum network lifetime) is a constraint of the problem; the goal focuses on the full exploitation of this residual energy. This design penalizes total cost, because all network nodes have to incorporate a renewable energy system supply. The work in [7] also proposes an optimal algorithm based on a finite time-horizon, which simultaneously maximizes throughput (defined as transmitted bitrate) and network lifetime. Nevertheless, in [7], renewable energy sources are not taken into consideration.

In the same line of power consumption optimization, the authors of [4] propose an *α*-lifetime optimization framework. They assume that (i) nodes have a variable supply of renewable energy and (ii) the network will be functional as long as an *α* portion of the nodes remain alive. This kind of optimization provides longer lifetimes for the selected *α* nodes by minimizing their respective energy consumption. This is achieved, because the (1 – α) unselected nodes are used to dispatch the network traffic, which extends the other nodes' lifetimes by reducing theirs significantly

Regarding the second group of works, in [8], lifetime maximization is addressed by allocating different capacity batteries to network nodes (which also implies a different role for the nodes in the network). In this study, the authors formulate the problem and demonstrate the improvement of the WSN lifetime. To this end, the work proposes an algorithm that efficiently solves the allocation of the most powerful chemical batteries as a function of the nodes' energy requirements. However, the contribution is only focused on a fixed number for each type of these batteries determined beforehand.

In this same context, but referring to the placement of nodes in the area covered by the network, the work in [9] proposes to maximize the lifetime, characterizing the energy consumption through a Measurement Square Error (MSE) algorithm. The obtained results are relevant when the sum of all power batteries is used as a goal of the optimization. In the work conducted in [10], lifetime is maximized when a sink node is placed at the center of the nodes deployment, achieving significant results as regards the balancing of node operation (mainly understood as the traffic flow assignment from any arbitrary node to each of its neighbors in coverage). Furthermore, a pseudo-optimal traffic load balancing algorithm is also proposed, showing that the sink is reached by a number of hops in the order of *O*(log *N*). Both studies do not include nodes without energy restrictions in their formulation (*i.e.*, nodes with renewable energy systems). The authors of [11] develop a two-level WSN, where the lower level nodes transmit data to micro-servers at the top level, providing connectivity between the sensors and a central base station. This work provides a framework that optimizes the positions and the initial energy assigned to the micro-servers. This approach increases network lifetime in comparison with the one achieved by another WSN with the same initial energy, but equally distributed among all network nodes. It should be noted, however, that the use of micro-servers increases the cost and complexity of the installation. In [12], the authors study lifetime in WSNs with Gaussian-distributed nodes in a two-dimensional space. The main contribution is the identification of the key parameters for lifetime maximization and the proposal of two location algorithms for the nodes. However, the results achieved only apply to Gaussian distributions of the network nodes location and cannot be easily extended to other deployment distributions

The authors in [13,14] advocate for the utilization of virtual backbones in which a few nodes are in charge of collecting data from the remaining network nodes and transmitting them to the sinks. In both approaches, chemical batteries power all the nodes, restricting the network lifetime in case of a single backbone. To solve this concern, [13,14] offer solutions based on complex algorithms, which schedule multiple overlapped backbones. In particular, during the network operation, these algorithms determine the nodes that establish the backbone as a function of metrics, such as the residual energy level. Thereby, these nodes get a new role within the network. Theoretical results obtained by the authors show that virtual backbones balance the network energy consumption among all sensor nodes, considerably increasing network lifetime. However, the real implementation of these algorithms in the nodes would entail, *a priori*, a higher number of control messages along with an increase in memory and processing demands; those issues are not quantified by the authors. It should be mentioned that these findings are consistent with our work, since optimal primary node placement produces topologies similar to virtual backbones (as we will see later in this paper).

Apart from the previous classification, a special case is the mobile sink proposed in [15]. The aim of this paper [15] is to maximize lifetime by solving a linear model, so as to choose which particular nodes the mobile sink must visit. As can be expected, a mobile sink increases network lifetime. However, the authors do not consider the energy required for sink mobility and other crucial issues, such as the technological feasibility of the solution. On the other hand, the work in [16–20] discusses several WSN cross-layer algorithms intended for lifetime maximization by using appropriate flow assignment techniques. These algorithms offer better lifetime than traditional solutions as the IEEE 802.15.4 standard. However, one of the major drawbacks is the centralized behavior of all of them, which, in practice, makes them unfeasible for large-scale networks.

Concerning WSN with renewable power sources and those that do not take into consideration LPtechniques, Niyato *et al.* [21] study the impact on packet transmission losses, by means of a game theoretic approach, when all network nodes incorporate solar-based energy harvesting systems. They obtain the packet loss probabilities, showing significant differences as a function of the solar energy captured. In addition, the authors optimize the duty cycle (the time period in which a node operates; the remaining time, the node is in the sleep mode, therefore, consuming less energy) of the nodes taking into account the packet loss probabilities previously calculated. These considerations are a particular case of our work, since it is intended for any renewable technology.

Finally, the work in [22–24] should be pointed out, because they provide practical algorithms that can also be implemented in sensor nodes (in particular, [24] implements a protocol tested in a real network/scenario), as well as a complete analytical study. Kim *et al.* [22] optimize two objectives in a network in which nodes are only fed by chemical batteries. Firstly, the authors minimize the packet delay metric in order to later maximize the lifetime. The result is a duty-cycle that thoroughly satisfies both optimization objectives. However, these results would clearly improve if the authors considered in its study nodes with renewable energy sources. On the other hand, the work in [23] maximizes the lifetime in a cluster topology. To this end, the algorithm calculates (i) the optimal one-hop distance to cover the maximum number of nodes and (ii) the optimum size of the cluster. With these two values, the algorithm selects the most appropriate cluster heads. The authors' solution also optimizes the residual energy value for the cluster head chosen, setting this value as a threshold. In the case that the threshold value is reached, the algorithm selects a neighbor of the cluster head as the new head. Unlike our proposal, [23] does not consider primary nodes and load balancing, because the traffic is always centralized by the cluster heads. Concerning the load balancing, an optimal lifetime solution based on traffic load and energy consumption is presented in [24]. The authors present two approaches. First, a traffic load balancing technique is used to optimize the energy consumption of the nodes in a grid topology with a base station in a corner. Secondly, a distributed heuristic algorithm extends the improvement of the traffic load balancing to any network topology. As in previous works, all the nodes have the same role and do not take advantage of renewable-energy harvesting systems.

Unlike all these works, we contribute with the estimation of the optimum number of nodes, incorporating renewable energy sources, maximizing network lifetime. In addition, our analytical model is able to obtain the precise location of these nodes into the network regardless of its topology. On the other hand, our research also optimizes the traffic load balancing between a node and its neighbors. These results enable a developer or researcher to easily carry out his own deployment/test-bed, beforehand, learning about (i) the specific nodes that must implement a renewable energy system and (ii) the traffic load share that a node must deliver to each neighbor.

## 3. Modeling and Formulation of the Problem

In this section, we develop a linear programming formulation for the optimal primary node and flow assignments in WSNs. Basically, we consider that a set, *P*, of primary nodes is optimal if no other set, *P*′, implementing an optimal network flow assignment scheme can provide a longer network lifetime. Our problem formulation consists of two steps: (1) lifetime optimization and (2) flow optimization.

In our model, we assume a connected WSN, *G*, of *N* nodes. We also assume that all nodes transmit information to a sink node in a hierarchical network and that any node can dispatch packets from other nodes. Two nodes are connected if the distance between them is less than or equal to the transmission range. Regarding the energy model, for simplicity, we assume that sink and primary node lifetimes are much longer than those of secondary nodes, *i.e.*, we approximate these lifetimes by ∞. Secondary node battery lifetime is limited, and once it is depleted, the node is considered dead. Network lifetime is defined as the minimum secondary node lifetime.

### 3.1. Linear Programming Formulation

The problem of primary node assignment is equivalent to that of maximizing network lifetime as shows Table 1. However, flow assignment optimization requires knowing the maximum network lifetime, as long as primary node assignment optimization only optimizes the flow assignment for the nodes dying first. The remaining nodes will be assigned any flow assignment scheme that guarantees that their lifetime is longer than or equal to the minimum network lifetime. For simplicity, we will consider node 1 as the sink for all scenarios.

Table 2 shows the parameters employed for the linear programming formulation. Expressions (1) to (6) are the linear problem constraints for the lifetime maximization problem:

- Expression (2) avoids any flow being lower than zero.
- Expression (3) means that all flows are transmitted to the sink.
- Expression (4) represents the flow conservation, *i.e.*, the summation of the outgoing flows minus the incoming flows of a node, *i*, must be equal to its data transmission rate.
- Expression (5) models the energy constraints of a node. Note that, in our model, a node consumes energy, even if it is in an idle state or not transmitting. When *I_i_* = 1, node *i* is considered a primary node, and we consider its energy to be virtually ∞.
- Expression (6) is used to limit the number of primary nodes.

It is important to stress that there may be several optimal primary node setups. Any of these solutions will maximize lifetime, but we will choose the one that also optimizes the network flow assignment. For this reason, we perform a second optimization step.

**Figure 1.**
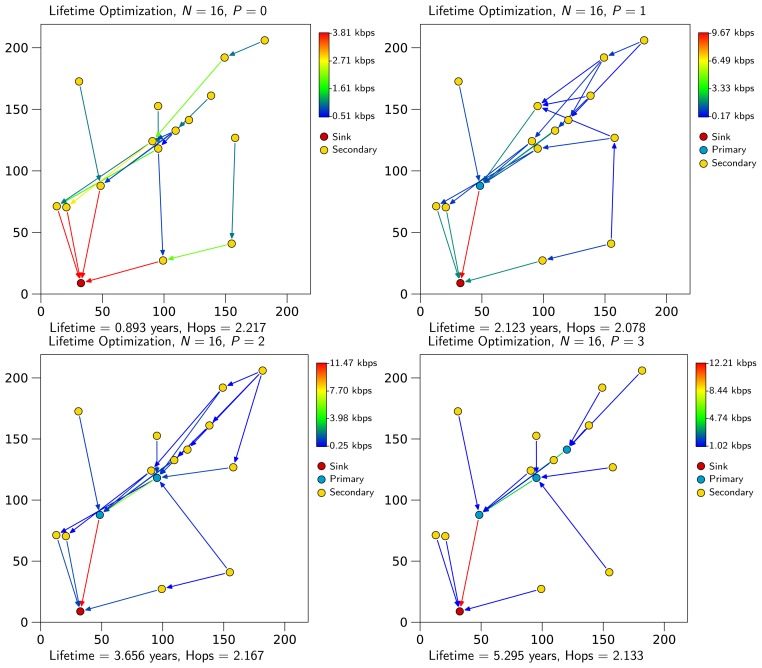
Lifetime optimization example results for a scenario with 16 nodes. Note how transmission schemes change with the assignment of new primary nodes. Link colors depend on the transmission rate according to the color bar on the upper right corner of each figure.

Figure 1 shows an example scenario for *N* = 16, in which primary nodes are represented in blue, secondary nodes in yellow and the sink in red. It can be observed that when no primary nodes are available, secondary nodes tend to dispatch traffic through several nodes. This way, traffic is not concentrated on a specific node. As primary nodes become available, they receive most of the traffic from other nodes. When lifetime is maximized, no secondary node relays traffic, as it would reduce its lifetime. Moreover, any optimal solution requires that any primary node can transmit packets through another primary node or transmit them to the sink. In this example, maximum lifetime is achieved with three primary nodes, something that happens when all secondary nodes can relay data through a primary one or send them directly to the sink. Figure 1 also represents the throughput in each link, measuring the amount of information per unit of time that reaches network nodes. As can be observed, the throughput in a link increases as the nodes under consideration (in particular, primary nodes form the virtual backbone) are closer to the sink.

The first optimization provides the maximum lifetime, *T_P_*, which canbe achieved when *P* primary nodes are available and the primary node assignment is solved, *i.e.*, *I_i_* = 1. *T_P_* will be used during the second optimization. However, during that optimization, it is possible to choose a different set of primary nodes, because, as pointed out before, there may be several solutions that maximize lifetime, but each one will have a different optimal flow assignment outcome (see Table 3). Thus, we choose the one providing the best flow assignment scheme, which we understand as the minimum average number of packet hops, as explained below.

Expressions (7) to (12) are the set of constraints for the flow assignment optimization problem:

- In this problem, we minimize Expression (7), which is the summation of products of the distance and the flow between all connected nodes. Minimizing flows is equivalent to minimizing the number of hops, since the average number of hops per packet, *H̅*, can be calculated as:
  (1) $$\bar{H}=\overset{N}{\underset{i=2}{\text{∑}}}\overset{N}{\underset{j=1}{\text{∑}}}\frac{f_{ij}}{r(N-1)}$$
  Distance *D_ij_* does not reduce *H̅*, but when there exists several feasible paths with the same minimum number of hops, the optimizer will choose one with the minimum total physical distance. Thus, removing *D_ij_* is possible, and it does not affect *H̅*.
- Expressions (8) to (12) are equivalent to Expressions (2) to (6). However, *T_P_* is now a constant obtained during the previous lifetime optimization.

After these two optimization steps, both lifetime and number of packet hops in the WSN are optimal. Moreover, the optimization process also provides a set of primary nodes to achieve the optimization goal.

**Figure 2.**
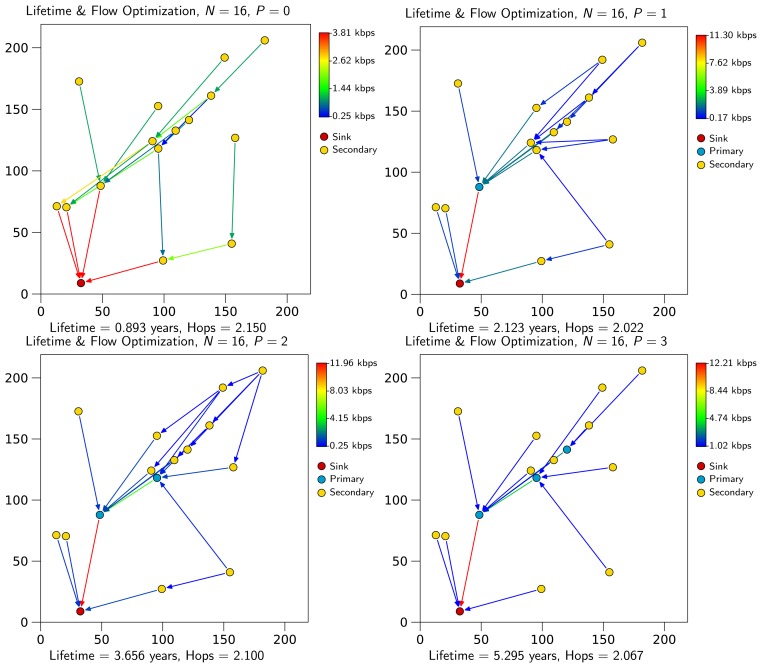
Flow assignment example for a scenario with 16 nodes (the same scenario as in the example of Figure 1).

Figure 2 shows the same example of Figure 1 after the second optimization step. It can be observed that flows get optimized in terms of the average number of hops (e.g., observe node 5 for *P* = 3 in Figure 1 and Figure 2). Note that increasing the number of primary nodes does not necessarily decrease *H̅*, as we prioritize lifetime over the number of packet hops. In general, adding primary nodes implies that secondary nodes relay packets to the closest primary node to the sink to save energy.

LP optimization is simple, although it has some scalability problems. The integer part of the problem implies that its complexity is $O\left(\left(\begin{array}{c} N-1\\ P \end{array}\right)\right)$, as this is the number of combinations of *P* primary nodes over *N* – 1 nodes. Thus, as *N* → ∞, the problem becomes unfeasible. To overcome this, let us define *A* as the network area and Δ as the node density, Δ = *N*/*A*. We propose to remove from the problem all the primary nodes that cannot provide an optimal solution. To this aim, for a low/medium density network, a primary node must be in the range of another primary node or the sink, as lemma 1 proves. Furthermore, any node with a coverage radius of *c* is connected on average to a number of nodes, *k* = *πc^2^*/Δ – 1; using this approach, we determine an upper bound for the complexity of the LP problem of *O*(*k^P^*). This approach is scalable, as it does not depend on *N*. However, it is also true that the number of required primary nodes to achieve the maximum lifetime grows with *N*, so *P* → ∞.

**Lemma 1**: In a WSN deployment, any primary node must be in direct coverage(*i.e.*, in range) of any other primary node or the sink if each network node satisfies at least one of the following premises: (i) to have, as a maximum value, five neighbors or (ii) to guarantee an amount of neighbors in the path to the sink smaller than half of the total neighbors.

**Proof:** The opposite sentence, a primary node may be unconnected to another primary node or the sink, is false.

Let us take a primary node (*P_x_*) unconnected to another primary node or the sink; it must necessarily dispatch their packets to M different secondary nodes (*S_1_*, *S_2_*, *S_3_*, …, *S_m_*). M takes a value between one and half of *P_x_*'s neighbors to fulfill the aforementioned lemma's premises. As all these nodes are not primary nodes, their consumed energy must be lower than the total consumed energy by the primary node, *P_x_*, during the network lifetime. Furthermore, any of these secondary nodes cannot be the first node to die, because in that case, the optimizer had assigned it as a primary node. Therefore, another secondary node of the network, *S_k_*, must be the first one to deplete its battery. In this scenario, as all nodes have the same data transmission rate, we define *E_λ_* as the minimum energy required by a node to transmit only the total information generated by this node (therefore, no computing relay/ retransmission tasks).

In addition, the following two sentences must be true: (i) the total energy consumed by node *P_x_* (*E_px_*) and, also, the wasted one by node *S_k_* (*E_sk_*) during the network lifetime are bigger than *E_λ_*, because *P_x_* and *S_k_* perform relay tasks and (ii) the *E_px_* must be greater than *E_sk_*, as node *P_x_* is primary In this case, *E_px_* = *E_sk_* + *θ*, where *θ* is the difference between both consumed energies. *E_sy_* is the power consumed by any *M* secondary node (denoted as *S_y_*) in coverage with the primary, including, on the one hand, *E*_λ_, due to the transmission of its own data transmission rate, and, on the other hand, the energy required by the node, *P_x_*, to dispatch a fraction/part of its traffic. In particular, $\frac{E_{P}x}{M}$ is the fraction of energy for the best case/scenario, because primary node *P_x_* balances the same amount of traffic among all the *M* secondary nodes in its coverage range. Obviously, the $\frac{E_{P}x}{M}$ value must be lower than (*E_sk_*), because secondary node *S_k_* depletes its battery before any other *S_y_*. Considering *M* > 1, this result implies that $E_{Px}>\frac{M}{M-1}E_{\lambda }+\frac{\theta }{M-1}$, as expressed by Equation (16) below, which is only feasible if *M* is a high enough value to relay packets of *M* + 1, that is, there are more secondary nodes between node *P_x_* and the rest of the network primary nodes than the secondary nodes associated with node *P_x_* to deliver their traffic through it. This result guarantees that the node, *S_k_*, depletes its battery before any other node, *S_y_*, restricting the design to the case of a **high-density node.** However, this design presents a double shortcoming: on the one hand, a clear increase of the deployment cost, and, on the other hand, the premise labeled as (ii) is not fulfilled. Finally, in the case of a few secondary nodes in coverage range with the primary, that is, a **low-density node deployment** (*M* = 1, 2, 3, 4), the outcome is unfeasible, because the optimizer would have to set one of these secondary nodes as a primary node, thus contradicting this proof.

(2) $$E_{Sx}=\frac{E_{Px}}{M}+E_{\lambda }<E_{Sk}$$

Substituting *E_Px_* = *E_Sk_*= + *θ* and operating:

(3) $$\frac{E_{Sk}+\theta }{M}+E_{\lambda }<E_{Sk}$$

(4) $$\frac{\theta }{M-1}+\frac{M}{M-1}E_{\lambda }<E_{Sk}<E_{Px}$$

### 3.2. Minimum Number of Primary Nodes

The most important design issue when maximizing network lifetime is to determine the minimum number of primary nodes that maximizes network lifetime. This number depends on the network node deployment, and we also know that all secondary nodes lie within the range of a primary one. Intuitively, it can be seen that a relation must exist between *N* and *P*. One approximation, without loss of generality, is to think of the network as a set of discs centered in the sink, with *N* being a large number. Thus, all nodes at a distance between *nc* and (*n* + 1)*c*, *n* ∈ ℕ, *n* ≥ 0 require at least *n* relays to reach the sink node. Moreover, to obtain a lower bound, a disc, *n*, requires at least *three*(*n* + 1) primary nodes to relay data from disc *n* + 1, *i.e.*, in the best case, if these relays are placed at the border of the disc at a distance of 2c. Consequently, a WSN network with *n* + 1 discs would require a minimum of *P_min_* = *3n*(*n* + 1)/2 primary nodes.

In our model, node density is constant, *i.e.*, the area is a function of *N*, *A* = Δ*N*. Thus, *π*(*nc*)^2^ ≥ *A*:

(5) $$n\geq \frac{1}{c}\sqrt{\frac{\Delta N}{\pi }}$$

If we plug Expression (17) in *P_min_*, we obtain a lower bound on the number of primary nodes as a function of *N*:

(6) $$P_{\min}\geq \frac{3\Delta N}{2\pi c^{2}}+\frac{3}{2c}\sqrt{\frac{\Delta N}{\pi }}\underset{N\to \infty }{⪆}\frac{3\Delta N}{2\pi c^{2}}=O(N)$$

Expression (18) indicates that the number of primary nodes to maximize lifetime grows linearly as *N* → ∞. Moreover, the complexity of the *P_min_* LP optimization is *O*(*k^N^*).

## 4. Primary Node Assignment Algorithm

Optimal primary node assignment complexity can be reduced to *O*(*k^P^*) by discarding all candidate combinations prior to optimization that will not maximize lifetime. However, as *P_min_* = *O*(*N*), if *N* → ∞, the LP optimization becomes unfeasible. To overcome this, we propose the OPT-PRIMalgorithm, which approximates the LP optimization based on the following assumptions:

- We assume that, for any solution, an appropriate network flow assignment given by a set of primary nodes maximizing lifetime can be found.
- The more secondary nodes within the range of primary nodes, the more network lifetime can be expected. However, this is not necessarily true, as lifetime is limited by the secondary node, dispatching more traffic.
- All primary nodes must be within the range of the sink node or another primary node (lemma 1). Moreover, from any primary node, there must exist a path of only primary nodes to the sink. If this condition is not accomplished, the solution cannot be optimal.

Algorithm 1 develops the pseudocode of OPT-PRIM. Table 4 is a summary of all variables used by OPT-PRIM. Algorithm OPT-PRIM is simple:

- OPT-PRIM is iterative. It ends when the maximum lifetime is achieved or the desired number of primary nodes has been allocated.
- Steps 1 and 2 are for initial setup and solution feasibility. If the network is unconnected, the algorithm ends.
- Step 3 updates some variables used in subsequent steps.
- In Step 4, the algorithm generates a list of primary node candidates based on the candidate list from the previous iteration. For each set of primary node candidates, the algorithm adds an extra node from the list of connected nodes. Thus, the set size grows in one unit. Note that Π^0^ = 1, *i.e.*, the sink node is treated as a primary node by the algorithm. This also guarantees that all primary nodes lie within the range of the sink or another primary node and that they can reach the sink directly or through another primary node.
- Step 5 removes redundancies to speed up the algorithm in subsequent iterations.
- Step 6 generates a list of nodes connected to each primary node candidate of that iteration that will be used in the next iteration. In Step 7, if the algorithm finds a set connected to all nodes in the WSN, it ends and labels those nodes as primary nodes.
- Step 8 goes back to Step 3 if the set size is not *P*.
- If Step 9 is reached, a list of candidates has been obtained. From that moment, the goal is to select the best set of candidates, as some secondary nodes will have to relay third party packets.
- In Step 9, the minimum number of hops to reach the farthest node in the WSN is obtained.
- Finally, in Step 10, the algorithm selects as primary nodes the set accomplishing the minimum number of hops to the farthest node. Thus, the algorithm aims at minimizing the amount of data dispatched by secondary nodes and, as a consequence, maximizing lifetime.

Section 5 presents some OPT-PRIM results. However, before ending this section, we would like to discuss some of its design issues:

- The algorithm is able to find an optimal solution in terms of network lifetime. We also know from the optimization framework that some node assignments allow implementing better flow assignment schemes. In the case of OPT-PRIM, primary node assignments are comparable to those of the first part of the optimization framework, which also focuses on lifetime optimization. Roughly speaking, the number of operations to allocate *P* primary nodes in OPT-PRIM is upper-bounded by *O*(*P*^2^*N*), P ≪ *N*, lower than $O\left(\left(\begin{array}{c} N-1\\ P \end{array}\right)\right)$ of the LP optimization.
- In the WSN model of this paper, network lifetime is limited by the lowest secondary node lifetime. In practice, secondary node lifetime decreases if the node has to dispatch traffic from third parties. Thus, as long as this can be avoided, network lifetime will increase. OPT-PRIM seeks to connect most secondary nodes to primary ones, but among all possible choices, it selects the one that minimizes the average number of hops from any secondary node to its closest primary node.

**Algorithm 1**: OPT-PRIM

Step 1For iteration *k* = 0, Π^0^ = 1, Γ^0^ = *ρ*_1_.Step 2Obtain ℋ*_ij_*. If max{ℋ*_ij_*} = ∞, ∀(*i*, *j*) ∈ *N*, then the WSN is unconnected and the algorithm ends.Step 3Update *k* = *k* +1, Π*^k^* = ∅, Γ*^k^* = ∅.Step 4For each row $\varrho_{i}^{k-1}$ in Γ*^k^*^–1^:
If $\gamma_{ij}^{k-1}=1$, then $\varphi =\varrho_{i}^{k-1}|p_{j}$.If $\varphi \neq \varrho_{i}^{k-1}$, aggregate *φ* to Γ*^k^* and vector $V=[\iota_{i}^{k-1}j]$. to Π*^k^*. Otherwise, remove node j.Step 5Remove duplicate rows in Π*^k^* (e.g., [1 7 2] and [1 2 7] are considered equal) and Γ*^k^*.Step 6For each row, $\varrho_{i}^{k}$, in Γ^k^, calculate $S_{i}=\underset{j}{\text{∑}}\gamma_{ij}^{k}$.Step 7If ∃*m* \ S*_m_* = *N*, the algorithm ends, and the selected primary nodes are $\iota_{m}^{k}$.Step 8If *k* ≠ *P*, go to Step 3.Step 9
$\forall S_{m}\backslash S_{m}=\underset{i}{\max}S_{i}$:
For each $\gamma_{mj}^{k}=0$, calculate ${\text{h}}_{mj}=\underset{\iota_{m}^{k}}{\min}{ℋ}_{jn}$.Calculate $H_{m}=\underset{j}{\max}h_{mj}$.Step 10Select $\iota_{n}^{k}$ as primary nodes if $H_{n}=\underset{m}{\min}H_{m}$.Step 11The algorithm ends.

## 5. Numerical Results

This section summarizes some relevant results and provides some examples that will help to understand the benefits of the optimization frameworks.

**Figure 3.**
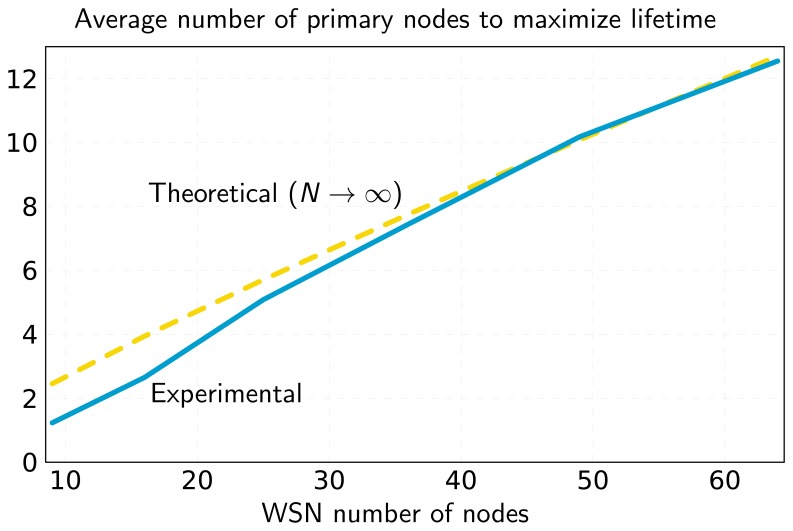
Comparison of theoretical and optimization results for the average minimum number of primary nodes required to maximize lifetime in a WSN.

Figure 3 compares the average number of primary nodes calculated by Expression (18) with the results from the optimization experiments. As can be seen, both average values converge as *N* grows. This validates our previous assumption that the minimum number of primary nodes grows linearly with *N*. Consequently, it is possible to calculate a rough estimate of the required primary nodes with the only knowledge of the number of nodes and the network area. From the point of view of network planning, the ability to estimate the number of primary nodes required to maximize lifetime speeds up the process of network design.

**Figure 4.**
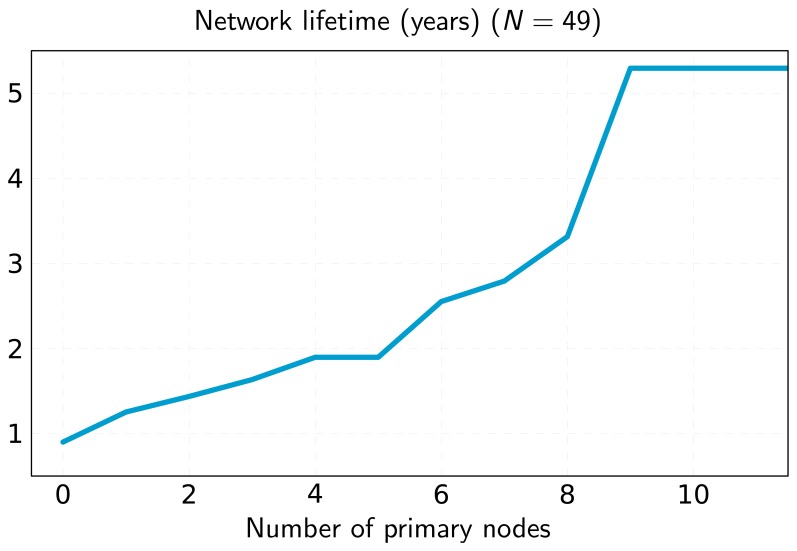
Network lifetime as a function of the number of primaries for the network of Figure 6.

**Figure 5.**
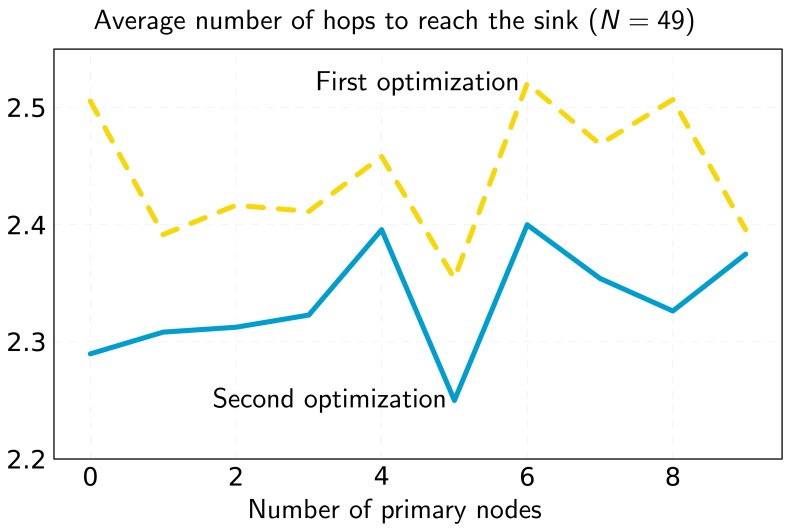
Minimum average number of hops for the *N* = 49 scenario of Figure 1 after the first and second optimizations.

Figure 4 represents the maximum network lifetime as a function of the number of primary nodes. It can be seen that adding primary nodes increases lifetime, although it may remain constant in some cases (e.g., for 4–5 primary nodes). This happens when there are several nodes with minimum lifetime. In this case, adding a primary node to the network will not help to increase lifetime. However, even if lifetime remains constant, this extra primary node can be used to optimize the flow assignments and reduce the number of packet hops, as can be observed in Figure 5.

Regarding flow assignment (average number of hops), Figure 5 shows that the addition of primary nodes provides different results leading to increments in some cases and reductions in others. However, the *general trend* is that the average number of hops increases with the number of primary nodes. As lifetime optimization has priority over *flow assignment optimization*, a node will choose a primary node rather than a secondary one, even if there exists a shorter path through the secondary node. Note, as a particular case, that when a primary assignment does not increase lifetime, it can be used to optimize flows, as it can be assigned to any node. This result is consistent with [25], where it is stated that there exists a tradeoff between lifetime and the average number of packet hops. Our scenario is slightly different, as we employ primary nodes and do not perform routing, but the idea behind it still holds.

**Figure 6.**
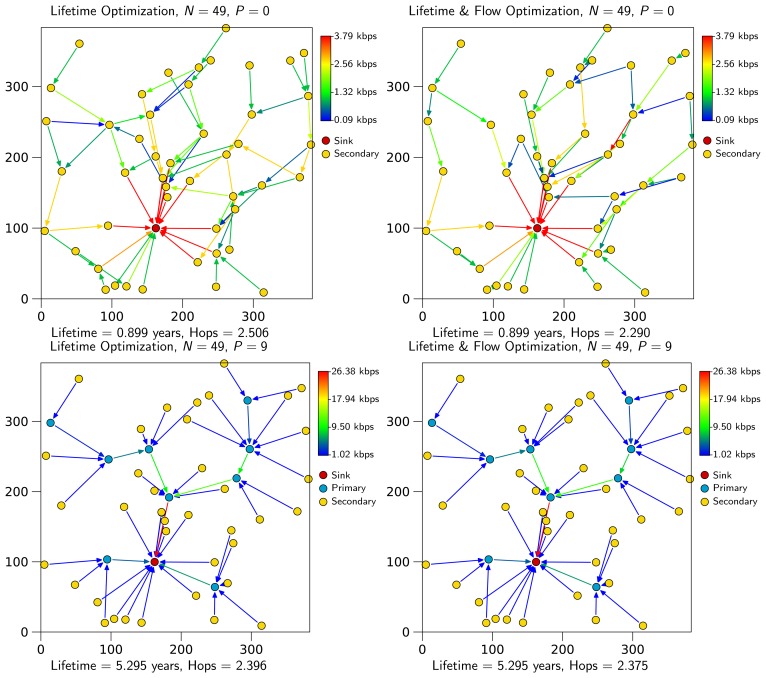
Example network of 49 nodes. Lifetime optimization without primary nodes. Top-right: lifetime and flow optimization without primary nodes. Bottom-left: lifetime optimization with nine primary nodes. Bottom-right: lifetime and flow optimization with nine primary nodes. Note that primary nodes increase network lifetime, whereas flow optimization reduces the average number of packet hops.

Some comments can be made regarding the particularity of the optimal primary node assignment. If we take Figure 6 as a reference, we can observe that the primary node assignment can be explained as a problem of building a virtual backbone to be able to transmit all network traffic. This kind of solution is optimal, because by concentrating all traffic in a few nodes (those that form the backbone), the number of primary nodes to maximize lifetime is minimal. This result is consistent with [13,14], a WSN architecture based on virtual backbones that considerably increases lifetime.

**Figure 7.**
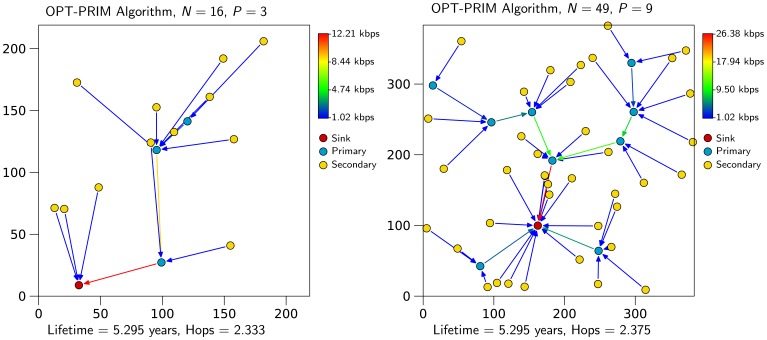
OPT-PRIM primary node assignment for the networks of Figure 2 and Figure 6.

**Figure 8.**
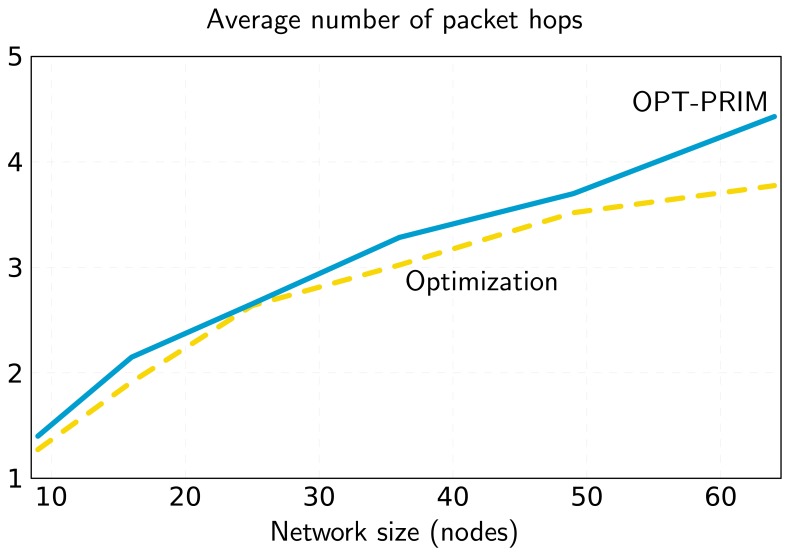
Minimum average number of hops achieved with OPT-PRIM and the optimization framework.

Regarding the primary node assignment algorithm, OPT-PRIM, the results are very similar to those of the optimization framework. However, note that the aim of OPT-PRIM is to maximize lifetime, but it does not consider the optimization of the average number of packet hops between any secondary node and the sink. Thus, a higher number of packet hops can be expected when using OPT-PRIM. Figure 7 shows the results obtained for the same scenarios of Figure 2 and Figure 6. Even though the average number of packet hops is larger for the 16-node network, for the 49-node example, OPT-PRIM achieves the same result. Moreover, Figure 8 depicts the minimum average number of hops that can be achieved with OPT-PRIM and the optimization framework. As can be observed, OPT-PRIM is slightly less efficient in terms of average number of hops, but it attains the same network lifetime and it outperforms the execution time of the LP formulation for the same problem.

## 6. Performance Evaluation

Computer simulation is also used to conduct a performance evaluation study under the same general scenarios and requirements proposed in the previous sections. The results here obtained confirm the validity and feasibility of the numerical values achieved by the former optimization process. To this aim, we programmed our energy saving model in an ns-2 (Network Simulation 2) simulation environment [26] to study the energy consumed for the different topologies under consideration. In ns-2, the most usual reference for WSN is the Zheng simulator [27]. The first Zheng simulator implementation evaluated the performance of a detailed environment under the IEEE 802.15.4 standard [5], especially regarding the CSMA-CA (Carrier Sense Multiple Access with Collission Avoidance) channel access control and its mechanism of retransmission of messages. From this first approach, we implemented the necessary ns-2 functions to assess the same scenarios proposed in the optimization section and, therefore, to fairly compare their respective results.

To perform our simulations, different hierarchical topologies with a variable number of nodes (from nine to 400 nodes) were selected from the layouts generated by the optimization study. The distance between nodes is less than or equal to 100 meters, and the maximum transmission range is set to 100 meters. Furthermore, the network coordinator (root) is the sink node appointed to receive all network data. The rest of the network nodes deliver information to the coordinator by constructing their connectivity matrices and generating path/s towards a primary node regarding the results provided by: (i) the optimization study and (ii) the OPT-PRIM algorithm. In this sense, the network nodes assigned by our algorithm as primary ones were designed with this same role in the simulation framework. Constant Bit Rate (CBR) traffic was used for all network nodes, consisting of one message delivered per second (one message/s) at 2.4 GHz for the ISM (industrial, scientific and medical) band (250 Kbps nominal transmission bitrate) with a message size of 127 bytes at the physical (PHY) layer. Traffic generated by network nodes was dispatched under the premises of maximum network lifetime and minimum number of hops calculated by the LP study and OPT-PRIM algorithm. Finally, as most large-scale WSN applications take place in outdoor environments, we use the free-space propagation model, which is a well-accepted approach for these scenarios.

**Figure 9.**
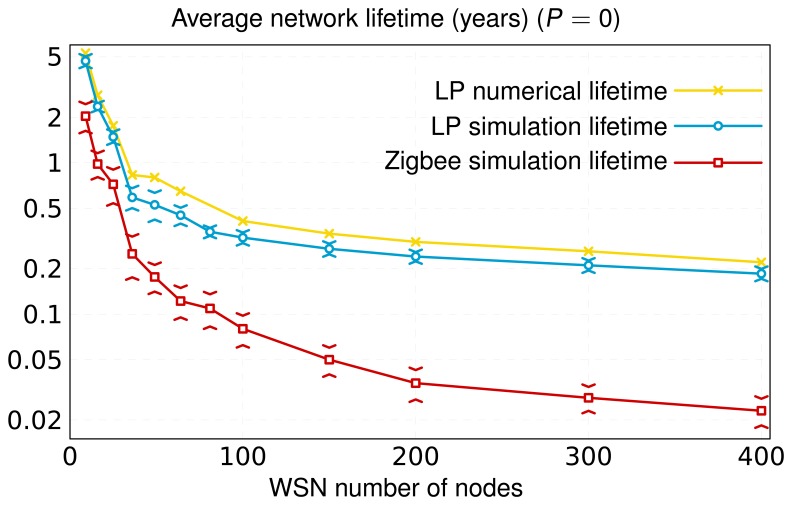
Network lifetime for different numbers of nodes without primary nodes. The simulation results show the average lifetime and its standard deviation obtained from 20 simulations with different seeds (95% confidence interval, Batch Mean Method).

In networks where the unique device without energy restriction is the network coordinator/root, the number of nodes has a significant impact on network lifetime. In this context, we have selected for Figure 9 the best results obtained among all the layouts analyzed by the optimizer in the case of zero primary nodes. The simulation results shown in Figure 9 reveal that networks with a high node density convey a reduction of network lifetime. The reason is twofold: (i) the increase in the number of messages disseminated by the network and (ii) the increase in the number of times that the CSMA-CA mechanism is triggered. As a consequence, there is extra energy consumption. When the network grows, the number of intermediate secondary nodes between the source and the sink (number of hops) also increases, which results in an augmentation of the number of messages dispatched and, consequently, of the probability of packet collision. To avoid packet collisions, IEEE 802.15.4 uses CSMA-CA. This mechanism is based on the idea that network nodes must wait for a random time (backoff time in the medium access protocol jargon) if the channel is busy. When a node is ready to transmit a message, it proceeds to listen to the physical medium, switching its transceiver to the ON mode. If the medium is free, the message is automatically transmitted, but if another neighbor device is also transmitting, the node aborts its transmission attempt and triggers the CSMA-CA algorithm. This algorithm defines the new time to attempt the transmission of the message under consideration. This process consumes energy, until the node finds free the medium, and as a result, the message is delivered. As a consequence, if the number of nodes grows, the probability that the channel is busy also increases, and therefore, more time and energy are invested in sending a message.

Following this line of reasoning, Figure 9 shows that networks of 400 nodes have a shorter lifetime than networks with fewer nodes. In this sense, note that in networks formed by a high number of nodes (large-scale) in which there are no primary ones, the inclusion of new nodes implies a slight reduction of the network lifetime in comparison with this same case applied to topologies with few nodes. In large-scale networks, there will be nodes with a large amount of neighbors in range, which increases the total traffic dispatched by all of them, and, therefore, the nodes' energy consumption by busy channel and data transmissions. Under these circumstances, nodes operate most of the time (they barely switch their transceiver to the OFF mode, thus saving energy), which sharply restricts their life. This is the explanation why the association of new neighbors almost does not entail additional operation by the nodes under study. However, networks consisting of a few nodes are clearly influenced by the appearance of new nodes, because more traffic will be dispatched and, therefore, more triggers of the CSMA-CA algorithm will be executed.

Figure 9 also includes the results for hierarchical Zigbee networks [28], the most extended commercial solution for WSNs. The Zigbee *de facto* standard runs continuously, updating the connectivity matrices of the entire network nodes to select the best (and unique) path between any arbitrary pair of source and destination nodes. Network nodes belonging to a particular source-destination path must receive all the data messages of their respective predecessor nodes and retransmit them. This procedure causes nodes included in an active path to consume more energy than the others, which leads to network node with a substantial energy disparity. This fact has a very negative impact on network lifetime. An appropriate redistribution of traffic flows by, for instance, selecting different paths for the same source-destination pair, as our algorithm does, is clearly advantageous in terms of energy saving. Thereby, the lifetime obtained for Zigbee is lower than our result.

**Figure 10.**
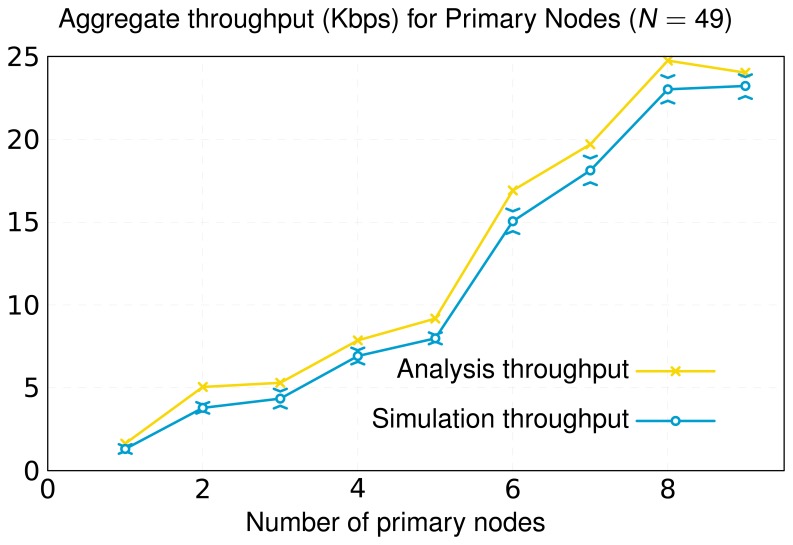
Aggregate throughput for primary nodes. The simulation results show the average aggregate throughput and its standard deviation obtained from 20 simulations with different seeds (95% confidence intervals, Batch Mean Method).

**Figure 11.**
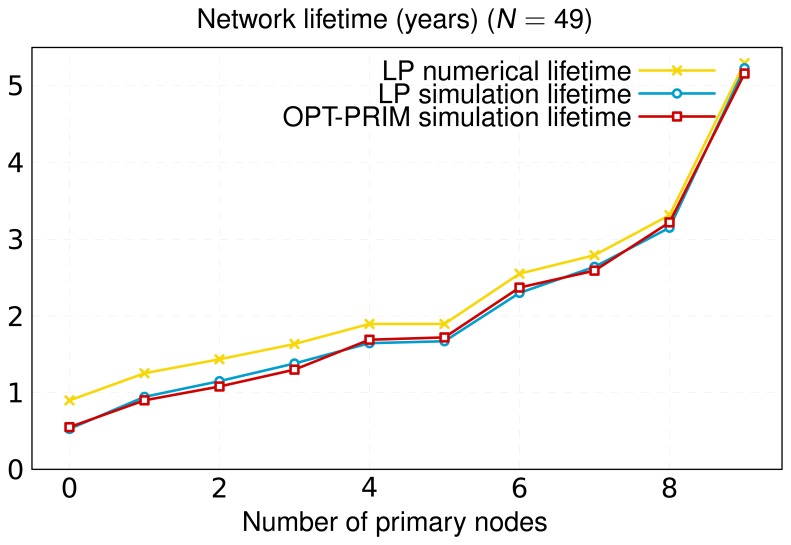
Average network lifetime as a function of the number of primary nodes for a WSN network of 49 nodes.

The inclusion of primary nodes in the network implies a redistribution of the traffic load. We quantify this issue by means of the metric aggregate throughput, which is represented in Figure 10. This performance metric applies to the primary nodes and characterizes the total bitrate (sum of all primary nodes' rates) dispatched by them. The simulation results obtained reflect that the consideration of new primary nodes and the appropriate selection of their locations yield a notable increase of the number of messages disseminated by these primary nodes. In our model, primary nodes have no energy restrictions, so that secondary nodes search for primary nodes to deliver their messages. This fact causes primary nodes to handle more data, alleviating the traffic load of secondary nodes. This way, secondary nodes consume less energy and, consequently, their chemical batteries operate for longer times. This contributes to a significant increase in the network lifetime, as shown in Figure 11. In this Figure, we illustrate the lifetime of a network composed of 49 nodes (same layout/scenario as that represented in Figure 6) as a function of the number of primary nodes. The results achieved corroborate that the increase in the number of primary nodes has a positive impact on the network lifetime. Furthermore, we can observe that a suitable layout of nine primary nodes generates a virtual backbone, where all the secondary nodes are placed to one-hop of at least one primary node. The result is a scenario that achieves similar lifetime to a network formed only by nine nodes without primary ones, where all of them are directly connected to the root (Figure 9). In addition, Figure 11 shows the simulation results for the two optimization approaches in this work: our optimization model based on linear programming and the novel OPT-PRIM algorithm. The network lifetimes obtained with OPT-PRIM are nearly the same as the outcome of the LP optimization model. This implies that the primary nodes selected by both techniques maximize network lifetime to the same extent. This validates that OPT-PRIM is an excellent alternative to LP optimization, especially for the cases where the problem formulation is complex or the time required to obtain a solution may be excessive.

Finally, in Figure 9Figure 10 and Figure 11, we also present the upper limit obtained from the optimization study. As can be seen, for the same topology, the lifetime and average throughput obtained by simulation is slightly less than the resulting one from the optimization analysis. This is mainly because the simulation includes the effect of the IEEE 802.15.4 MAC (Medium Access Control) and PHY layers. Thereby, for the same scenario, and according to the simulation, nodes consume more energy and yield less throughput, since the optimization procedure obviates real processes, such as channel access control or messages retransmission. However, simulation results are very close to the numerical ones, therefore validating the LP optimization study and the OPT-PRIM algorithm proposed.

## 7. Conclusions

Optimal planning of WSNs is a complex problem, due to the high degree of flexibility of this kind of network. For this reason, it is desirable to have tools that support this task. Moreover, as planning is usually based on simplified network models, the reliability of planning tools is very important. This paper has presented an optimization framework to simultaneously maximize lifetime and minimize *the average number of packet hops* for WSN networks. Additionally, we have also designed an algorithm that achieves results comparable to an optimization problem. In both cases, we have considered two types of nodes: nodes with *long-lasting batteries, or primary nodes*, and nodes with regular batteries, or secondary nodes. The goal of our optimization framework and the assignment algorithm is to deploy primary nodes, so that lifetime is maximized.

The optimization framework consists of two LP problems. The first one defines a set of constraints to maximize lifetime, whereas the second one is designed to optimize *flow assignments*, minimizing the average number of packet hops, once the maximum lifetime is known.

As the optimization framework is based on a simplified model, we have also performed some simulations of more realistic networks to validate the optimization results. Simulation results exhibit a close similarity with those of the optimization, which suggests that the simplified network model in the optimization framework is valid. Simulations also show that the well-known Zigbee standard provides poor results in comparison with our studies. Among other reasons, it does not distinguish between different node energy behaviors.

The optimized solution is consistent with previous works: Optimal primary assignment tends to form virtual backbones, which, as previously noted in [13,14], allows the increase of lifetime. Moreover, there exists a tradeoff between lifetime and the average number of packet hop efficiency. However, when lifetime cannot be increased, primary node assignment would permit an efficient network flow assignment, once lifetime becomes irrelevant.

Due to the complexity of the LP formulation, we also propose an optimization algorithm, OPT-PRIM, which achieves comparable lifetimes as those of the optimization. However, regarding the number of packet hops, it is slightly less efficient, but as OPT-PRIM is much faster, it can be considered a good alternative to complex or large-scale problems. Finally, we also have validated the results of the OPT-PRIM algorithm by means of simulations.

As future work, particular cases may be studied by the consideration of additional features related to the link level, routing scheme or application layer in the constraints of the optimization problem. This may lead to a more reduced solution space, reaching optimal results and narrowing the gap to the simulation results obtained by our study. On the other hand, feasibility/compatibility studies between our OPT-PRIM proposal and WSN routing algorithms, such as ALBA-R (Adaptive Load-Balanced Algorithm, Rainbow version) [29] or XLP (Cross Layer Protocol) [30], might mean the enhancement of network lifetime and other network performance figures, such as end-to-end delay or packet delivery ratio.

## Figures and Tables

**Table 1.** Problem formulation of primary node assignment.

**Primary node assignment problem constraints.**	
maximize	(1) $$\begin{array}{c} T_{P} \end{array}$$
subject to	(2) $f_{ij}\geq 0,\forall (i,j)\in N,i\neq j$
	(3) $$\overset{N}{\underset{j=1}{\text{∑}}}L_{j1}f_{j1}=(N-1)rT_{P}$$
	(4) $$\begin{array}{l} \overset{N}{\underset{j=1}{\text{∑}}}L_{ij}f_{ij}-\overset{N}{\underset{j=2}{\text{∑}}}L_{ji}f_{ji}=rT_{P}\\ \forall i\in N,i\neq 1,i\neq j \end{array}$$
	(5) $$\begin{array}{l} E_{tx}\overset{N}{\underset{j=1}{\text{∑}}}L_{ij}f_{ij}+E_{tx}\overset{N}{\underset{j=2}{\text{∑}}}L_{ji}f_{ji}+E_{c}T_{P}\leq \\ \leq E_{i}+MI_{i},\forall_{i}\in N,i\neq 1,i\neq j \end{array}$$
	(6) $$\overset{N}{\underset{i=2}{\text{∑}}}I_{i}\leq P$$

**Table 2.** Primary node assignment problem parameters.

**Parameter**	**Description**
*T_P_*	WSN maximum lifetime for *P* primary nodes.
*N*	Number of nodes of the WSN.
*P*	Number of primary nodes of the WSN.
*L_ij_*	If *L_ij_* = 1, there exists a link between *i* and *j*, otherwise *L_ij_* = 0.
	Additionally, the network is symmetric, *L_ij_* = *L_ji_*.
*D_ij_*	It represents the physical distance between node i and node *j*, *D_ij_* = *D_ji_*.
*f_ij_*	The data flow from *i* to *j*, *f_ij_* ≥ 0.
*I_i_*	A binary variable; if *I_i_* = 1, node *i* is a primary one.
*r*	Node transmission data rate.
*M*	A very large number, *M* ≫1.
*E_rx_*	Energy consumption to receive a packet.
*E_tx_*	Energy consumption to transmit a packet.
*E_c_*	Activity energy consumption (per time unit).
*E_i_*	Initial node *i* energy.

**Table 3.** Flow assignment optimization.

**Network flow assignment problem constraints.**	
minimize	(7) $\overset{N}{\underset{i=2}{\text{∑}}}\overset{N}{\underset{j=1}{\text{∑}}}L_{ij}D_{ij}f_{ij}$
subject to	(8) $f_{ij}\geq 0,\forall (i,j)\in N,i\neq j$
	(9) $$\overset{N}{\underset{j=1}{\text{∑}}}L_{j1}f_{j1}=(N-1)rT_{P}$$
	(10) $$\begin{array}{l} \overset{N}{\underset{j=1}{\text{∑}}}L_{ij}f_{ij}-\overset{N}{\underset{j=1}{\text{∑}}}L_{ji}f_{ji}=rT_{P}\\ \forall i\in N,i\neq 1,i\neq j \end{array}$$
	(11) $$\begin{array}{l} E_{tx}\overset{N}{\underset{j=1}{\text{∑}}}L_{ij}f_{ij}+E_{rx}\overset{N}{\underset{j=2}{\text{∑}}}L_{ji}f_{ji}+E_{c}T_{P}\leq \\ \leq E_{i}+MI_{i},\forall i\in N,i\neq 1,i\neq j \end{array}$$
	(12) $$\overset{N}{\underset{i=2}{\text{∑}}}I_{i}\leq P$$

**Table 4.** OPT-PRIM algorithm parameters.

**Parameter**	**Description**
*N*	Number of nodes in WSN *G*.
*P*	Number of primary nodes in WSN *G*, *P* < *N*.
*Λ*	Link matrix of *G* (one if true, zero otherwise).
*λ_ij_*	The element, (*i*, *j*), in Λ, *λ_ij_* = *λ_ji_*.
*ρ_i_*	Row *i* in matrix Λ.
ℋ*_ij_*	The number of hops of the shortest path from *i* to *j*.
Π*^k^*	The list of primary candidates after iteration k; it is a matrix of size *t* × *k* + 1, *t* ≥ 1.
$\iota_{i}^{k}$	Row *i* in matrix Π*^k^*.
Γ*^k^*	A matrix of nodes connected to the nodes in Π *^k^*. Each row in Π *^k^* corresponds to a row in Γ*^k^*.
$\gamma_{ij}^{k}$	The element, (*i*, *j*), in Γ*^k^*.
$\varrho_{i}^{k}$	Row *i* in matrix Γ*^k^*.
